# Anticipated benefits and challenges of implementing group care in Suriname’s maternity and child care sector: a contextual analysis

**DOI:** 10.1186/s12884-023-05904-y

**Published:** 2023-08-18

**Authors:** Nele Martens, Ashna D. Hindori-Mohangoo, Manodj P. Hindori, Astrid Van Damme, Katrien Beeckman, Ria Reis, Mathilde R. Crone, Rianne RMJJ van der Kleij

**Affiliations:** 1 Leiden University Medical Center, Leiden, the Netherlands; 2 Foundation for Perinatal Interventions and Research in Suriname (Perisur), Paramaribo, Suriname; 3 Department of Public Health, Vrije Universiteit Brussel (VUB), Jette, Belgium; 4 Department of Nursing and Midwifery Research Group (NUMID), Universitair Ziekenhuis Brussel (UZ Brussel), Jette, Belgium; 5 Centre for Research and Innovation in Care, Universiteit Antwerpen, Antwerp, Belgium; 6 Leiden University Medical Centre, Leiden, Netherlands; 7 Amsterdam Institute for Global Health and Development (AIGHD), Amsterdam, Netherlands; 8 University of Cape Town, Cape Town, South Africa

**Keywords:** Group care, Maternity care, Antenatal care, Postnatal care, Context analysis, Implementation, Implementation science, Consolidated framework for implementation research (CFIR), Global maternal health, Rapid qualitative inquiry, Suriname, SamenZwanger

## Abstract

**Background:**

Suriname is a uppermiddle-income country with a relatively high prevalence of preventable pregnancy complications. Access to and usage of high-quality maternity care services are lacking. The implementation of group care (GC) may yield maternal and child health improvements. However, before introducing a complex intervention it is pivotal to develop an understanding of the local context to inform the implementation process.

**Methods:**

A context analysis was conducted to identify local needs toward maternity and postnatal care services, and to assess contextual factor relevant to implementability of GC. During a Rapid Qualitative Inquiry, 63 online and face-to-face semi-structured interviews were held with parents, community members, on-and off-site healthcare professionals, policy makers, and one focus group with parents was conducted. Audio recordings were transcribed in verbatim and analysed using thematic analysis and Framework Method. The Consolidated Framework for Implementation Research served as a base for the coding tree, which was complemented with inductively derived codes.

**Results:**

Ten themes related to implementability, one theme related to sustainability, and seven themes related to reaching and participation of the target population in GC were identified. Factors related to health care professionals (e.g., workload, compatibility, ownership, role clarity), to GC, to recipients and to planning impact the implementability of GC, while sustainability is in particular hampered by sparse financial and human resources. Reach affects both implementability and sustainability. Yet, outer setting and attitudinal barriers of health professionals will likely affect reach.

**Conclusions:**

Multi-layered contextual factors impact not only implementability and sustainability of GC, but also reach of parents. We advise future researchers and implementors of GC to investigate not only determinants for implementability and sustainability, but also those factors that may hamper, or facilitate up-take. Practical, attitudinal and cultural barriers to GC participation need to be examined. Themes identified in this study will inspire the development of adaptations and implementation strategies at a later stage.

**Supplementary Information:**

The online version contains supplementary material available at 10.1186/s12884-023-05904-y.

## Background

Antenatal care (ANC) and postnatal care (PNC) are important pillars and indicators of public health. Traditionally ANC and PNC are delivered on a one-on-one basis. In contrast, group care (GC) is an innovative approach in which (expecting) mothers/couples (and infants in PNC) come together for up to ten two-hour sessions consisting of: 1) a health assessment conducted by health care professionals (HCPs), 2) self-assessments conducted by parents and 3) group discussions facilitated by HCPs [1, 2]. GC may enhance the parent-provider relationship, improve health behaviours and foster social support from peers [3]. Moreover, evidence points to promising effects on birth weight and preterm birth rates [4–8]. Due to the reported benefits and aiming to improve utilization and quality of ANC, the World Health Organization (WHO) recommends to broaden the implementation of GC globally [9].

Implementation refers to the extent to which an intervention is put into practice as intended [10, 11]. Various aspects of implementation can be distinguished and evaluated after, or prior to the introduction of an innovation. For instance, implementation delivery can be evaluated *afte*r the innovation is put into practice, whereas contextual factors can be explored to assess implementability—the likelihood that an innovation will be delivered—*before* introducing it in a new context [11].

The concept of context encompasses more than merely the setting. Context is the multi-layered set of dynamic characteristics and circumstances influencing implementation [10]. Complex interventions, such as GC, are prone to implementation failure when transferred to another context. If implementation fails, potential health benefits may not be attained rendering allocated resources futile [11–13]. Therefore, prior to implementing GC in different contexts, implementability should be examined through context analyses [14]. The Consolidated Framework for Implementation Research (CFIR) [15, 16] is frequently used to systematically study implementation determinants [17]. This theoretical framework comprises five domains, namely outer setting and inner setting in which the intervention is implemented, intervention, individuals involved in the implementation and the implementation process.

Insights gained from context analyses can inform intervention adaptations and implementation strategies that fit local needs, ultimately fostering implementation success [17, 18]. Context analysis, however, is often omitted due to limited resources and lack of methodological guidance [17]. Amongst others, these factors might also explain why implementation science is neglected in the field of maternity care research, yet much needed, [19] especially in low and-middle income countries with high Maternal Mortality Ratios (MMRs).

Suriname, a former colony of the Netherlands, is an upper-middle-income country in South-America with a population of about 583,000 inhabitants and a MMR that has plateaued over the last ten years at an average of 130 maternal deaths per 100, 000 births, of which ca. 50% are preventable [20]. Primary health care is delivered by three different health care providers. The Regional Health Service (RGD) is a governmental institution with ca 60 clinics in the coastal area. Usually, the RGD team consists of several nurses, midwives and general practitioner (GPs). A GP is the head of the clinic. Next to RGD clinics, private GPs operate in the coastal area, whereas the Medical Mission Primary Health Care Suriname (MMPHCS), a semi-governmental health care provider, operates exclusively in remote areas in the hinterlands. ANC in Suriname is based on WHO guidelines of minimal eight visits, preferably to start during first trimester of pregnancy, followed by two visits during the second trimester and five visits during the third trimester when no pregnancy complications occur. Although the Surinamese health care system officially follows the WHO guidelines, only 44.1–57.9% of pregnant women have at least eight ANC visits [21]. Only 56% of pregnant women had an ANC visit during the first trimester and thirteen percent of women delivering received no ANC [21]. Delayed ANC and increased risk of obstetric complications are linked to barriers in obtaining a health insurance cards [21, 22].

ANC is mainly provided in primary health clinics by midwives and GPs from the RGD or by private GPs, and in the interior by skilled health care workers under supervision of remote doctors in the capital city Paramaribo. Pregnant women with complications and those who plan to give birth at the hospital (the latter around week 32) are referred to gynaecologists or midwives at the hospitals. Almost all births in Suriname are supervised by a skilled HCP. Around ninety percent of births are hospital-based under supervision of a midwife or gynaecologist [21]. The remaining births take place under the supervision of a midwife or general practitioner at the RGD or supervised by a skilled healthcare worker at MMPHCS. Less than one percentage of births occur at home or elsewhere (e.g., during transport) without supervision of a HCP [21].

In 2014, three hospitals in Paramaribo introduced the GC model *SamenZwanger* as part of the Perinatal Interventions Suriname project funded by the Ministry of Foreign Affairs of the Netherlands through the Twinning Facility Suriname Netherlands [23]. After the project period *SamenZwanger* was continued in one hospital by *the Foundation for Perinatal Interventions and Research in Suriname* (Perisur); ten groups were conducted during the period 2017–2019. From 2019, *SamenZwanger* was continued in the hospital setting with payment from the participants. In an approach to make *SamenZwanger* sustainable and reach vulnerable women from deprived areas, midwives from the RGD were trained to facilitate GC in 2019 with funding from the *Pan American Health Organization*. In February 2020, the first RGD clinic implemented *SamenZwanger* at RGD Santodorp and in March two more clinics (Geyersvlijt and Latour) followed. Due to the Covid-pandemic all groups stopped, and no group sessions were organized during 2020–2022.

The Committee for Maternal Mortality Suriname (MaMS), recommends a multitude of measures to lower the MMR, including assessment of family and community care needs, preventative programs targeting vulnerable groups, and psycho-social support during and after pregnancy [20]. In line with the MaMS’ recommendations, GC will be implemented in four primary care settings located in disadvantaged sub-urban areas surrounding Paramaribo, the capital city of Suriname. A context analysis was conducted to identify local needs, gain insight into the standard maternity care services, and to assess implementation barriers and facilitators in the settings that were selected as pilot sites. The CFIR guided the context analysis [15, 16].

This context analysis seeks to answer two research questions: *(1)* What is the current situation of maternity care in Suriname?, and *(2)* What are the contextual factors relevant to the (continued) implementability of GC in Suriname?

## Methods

### Study design and setting

This study is part of the Horizon2020 project Group Care during the first 1000 days (GC_1000) [24]. GC_1000 aims to implement and scale-up contextually sensitive formats of GC in seven countries and to evaluate implementation processes. Prior to the introduction of GC in the selected settings, a Rapid Qualitative Inquiry (RQI) was conducted in order to study contextual factors relevant to the implementability of GC. Approval from the director of Ministry of Health in Suriname was attained on 26^th^ of January 2021.

Suriname can be divided into three distinct areas, based on geographic, socio-economic, and cultural characteristics: the urban coastal area, the rural coastal area, and the rural interior [25]. Two-third of the Suriname population (66%) is concentrated in the two largest, mainly urban districts: the capital Paramaribo and Wanica. The primary economic focus is on trade and small industries, and companies engaged in food production and processing, and other products for the domestic market [25]. The largest ethnic groups are Creoles and Hindustanis [25]. Four RGD clinics in the urban districts capital Paramaribo and Wanica were selected as pilot sites by an implementation team from the RGD and Perisur. Selection criteria included a suitable space for the group care sessions, at least two midwifes working at the sites for GANC and at least two nurses and at least two doctors for the sites for postnatal GC, number of women receiving ANC and mothers/babies receiving PNC large enough to create groups for ANC and PNC.

### Participants and sampling

Purposive sampling as described by Tongco [26] was employed with reliance on the Perisur network in order to recruit respondents from the outer context (policy makers/advisors, external healthcare professionals and NGO employee), whereas on-site HCPs, recipients and community members were purposively sampled at, or via the implementation sites. Women who participated during 2017–2020 in the *SamenZwanger* groups were invited to participate in a focus group discussion. All respondents were informed about the GC_1000 study and if they consented to participate in writing or verbally, an interview was scheduled.

### Data collection

An RQ) took place in March and April 2021. RQI is a time and cost-effective, team-based technique that focuses on insiders’ perspectives and uses triangulation and iterative data analysis to gain preliminary understandings of complex situations [27]. In collaboration with local researchers 64 online and face-to-face semi-structured interviews were conducted with care recipients, community members, on-site healthcare professionals (HCPs), policy makers/advisors, external healthcare professionals and one NGO employee, and one online focus group with recipients. While Surinamese researchers conducted face-to-face interviews, the external researchers from the Netherlands and Belgium were not able to travel due to covid restrictions and therefore they interviewed respondents online. The CFIR guided the development of three generic interview guides for *(1)* recipients, *(2)* HCPs, and *(3)* other stakeholders (see appendix 1–3 and Table 1), which were pre-tested and used for preceding RQIs in other countries that participate in GC_1000 (namely Belgium, The Netherlands, Kosovo, The United Kingdom, South Africa and Ghana). Semi-structured interview guides consisted of two parts. In the first part of the interview the current situation of maternity care and characteristics and needs of the target population were explored. For example, HCPs were asked *Can you describe the care trajectory for a pregnant woman?* and recipients *Can you describe your experience with antenatal care?*. Subsequently, a four-minute video introduced GC, followed by questions on the perceptions of GC and its implementability, such as *What do you think about this form of care?* and *How can successful implementation be ensured? What do you need?* (see Appendix 1–3). In contrast, the focus group guide was not divided in two sections and no explanatory video was used as respondents had participated in GC previously. The focus group was conducted in Dutch language and it took 120 min. The length of the interviews ranged from 15–100 min with a mean of 42 min and standard deviation of (SD) of 17 min. The majority of interviews were in Dutch (*n* = 57); four were in English and two in Sranan Tongo. Flexibility with regards to usage of interview guides and tailoring of questions to the experience, or expertise of interviewees was encouraged. During daily debriefings attended by local and external researchers the findings were pre-analyzed and further data collection needs/data saturation were discussed [26].

### Data analysis

All data sources were used to answer our two research questions. Audio recordings of interviews and the focus group were transcribed in verbatim and analyzed using thematic analysis [28]. The CFIR [3] served as a base for the coding tree and it was complemented with inductively derived codes. Constructs from all five CFIR domains served as codes and where later grouped into themes using the Framework Method [29]. Matrices where rows correspond to respondents and columns to codes allowed for reduction of data and comparison of what was said by whom. This facilitated the grouping of multiple codes into fewer overarching themes. To illustrate, the codes ‘GC format and outcome expectancy, ‘content’ and ‘group composition’ merged into the sub-theme ‘innovation’, which in turn forms part of the theme ‘implementability’. Coding was performed with *Atlas.ti 22* software by NM. Intercoder reliability was not sought as such quality insurance measures do not correspond to our epistemological understanding of qualitative research [28, 30, 31]. However, reflexivity and the interpretation of data were constantly discussed within the diverse research team [31] to ensure trustworthiness of results [31].

## Results

### Basic characteristics of the study participants

Table 1 provides definitions of the respondent categories and number of respondents per data collection method.

**Table 1:** Respondents

Category	Number	Description	Age (in years)	Gender	
				**Male**	**Female**
Recipients	34	FGD with SamenZwanger participants	range 18–48	0	7
		Pregnant women/mothers and partners/fathers (*n* = 9)	mean 30; SD 8	8	19
Community members	6	Prominent members of the communities surrounding implementation sites	not recorded	4	2
On-site HCPs	16	GPs (*n* = 4), midwives (*n* = 7), and nurses (*n* = 5) at the four implementation sites	not recorded	4	12
Policy makers/advisors	4	Policy makers and advisors in the health care sector	not recorded	1	3
HCPs	11	HCP professionals in Suriname who are not directly involved in GC_1000. Mainly GPs, specialized doctors (gynaecologist, pediatrician), and midwives (one with SamenZwanger experience)	not recorded	4	7
NGO employee	1	NGO focused on sexual and reproductive health	not recorded	0	1
**Total**	**72**			**21**	**51**

Contextual information on the current situation of maternity is provided below. Moreover, ten themes related to implementability, one theme related to sustainability, and seven themes related to reaching and participation of the target population in GC (i.e., pregnant women/couples and young parents) were identified. Whereas some themes and sub-themes can be directly linked to the CFIR (e.g., implementability, innovation design), others were inductively derived (e.g., reach, perceived necessity).

### Current maternity care

For women without pregnancy complications, ANC is provided at the RGD until ca. week 30 when they are referred to secondary care. The large majority of women give birth at the hospital, although delivery at the RGD is possible for low-risk multiparous women. Well-baby clinics, or *Consultatie bureau* (CB), are financed by the government and they are part of the RGD and MMPHCS.

A gap in health care usage/delivery in the first days and weeks postnatally was identified based on responses from multiple recipients, HCPs and on-site HCPs. RGD staff do not know when women deliver at the hospital and when they return to their homes. Therefore, midwives do not visit these mothers at home and for many the first contact postnatally with the health care system is for their child’s first vaccination (at eight weeks). HCPs explained that CB largely focuses on the infant, and a policy advisor stressed the need to monitor women’s health more closely postpartum. Moreover, several HCPs lamented that during CB consultations, the infants’ physical health is attended to exclusively, while the cognitive, emotional and social developmental assessments are neglected. Using the Bayley test [32] was suggested, an instrument to assess the motor skills, cognitive, language, and socio-emotional and behavioural development of babies.

*“So because, for example, an RGD midwife does home visits after the birth. But only to the people who gave birth with them. So if a woman gives birth in 's Lands Hospital, the midwife of the RGD clinic where she lives will not visit her at home. She only goes to those women who give birth there.”* Interview with HCP, ‘s Lands Hospital.

#### Paternal role

Respondents across categories explained that involvement of fathers in ANC and PNC remains low despite a noticeable increase. Interviewees argued that a hypermasculine culture, work obligations and marital conflict can explain the fathers’ absence. Low SES, being from the interior and young maternal age were also linked to absence of fathers. Care recipients and on-site HCPs thought that fathers should be encouraged to accompany mothers to health care appointments. However, one HCP pointed out that it was important to also consider other support people (such as mothers and aunts) in view of the typically variable family constellations in Suriname.

In Table 2 contextual factors relevant to implementability, sustainability and reach are summarized.

**Table 2:** Implementability, sustainability and anticipated reach

Themes	Sub-themes
**Implementability**	**Innovation**
	**GC format and outcome expectancy.** Several HCPs expected improved pregnancy outcomes and a lower perinatal mortality
	**Content.** Different views on herbal medicine and traditional remedies may surface during GC discussions
	**Group composition.** Ambivalence regarding diversity with regard to SES, ethnicity and inclusion of fathers
	**Multi-sectorial approach.** As determinants of neonatal and maternal health are diverse, a multi-sectorial approach is needed
	**Recipients**
	**Self-efficacy.** Self-efficacy to conduct self-assessments of some recipients is low and HCPs are ambivalent about self-assessments
	**HCPs**
	**Staff shortage and high workload.** Staff shortage and high workload prevail in the health care sector
	**Compatibility with working routine and motivation.** Despite eagerness to start GC, on-site HCPs were uncertain about time-effectiveness of GC and compatibility with their working routine
	**Buy-in and ownership.** Buy-in from RGD managers, RGD HCPs and other professionals in the field was high
	**Role clarity.** Roles of different professionals involved in the GC project are not clearly defined
	**Planning**
	**Planning and logistics.** It is unclear when and where GC session will take place
**Sustainability**	**Economic situation.** Sparse resources are allocated to curative and not to preventative care
**Reach**	**Outer setting**
	**Competing demands.** Many parents in the sub-urbs of Paramaribo experience economic stress and difficulties with their health insurance
	**(Care) infrastructure**. Disparate access to care, esp. disadvantageous in the interior
	**Social environment.** Romantic/family relationships and power dynamics are complex and gender inequality prevails
	**Acceptability**
	**Cultural sensitivity.** Cultural traditions and beliefs differ between the various regions and ethnic groups, and they may interfere with health seeking behaviours
	**Perceived necessity of care.** Preventative care is frequently not considered necessary
	**Marketing and communication.** Some recipients though that GC targets couples of high SES
	**Privacy, confidentiality and trust.** Implementation sites are nested in a tightly-knit communities where fear of judgement is high

### Implementability

**Fig. 1:**
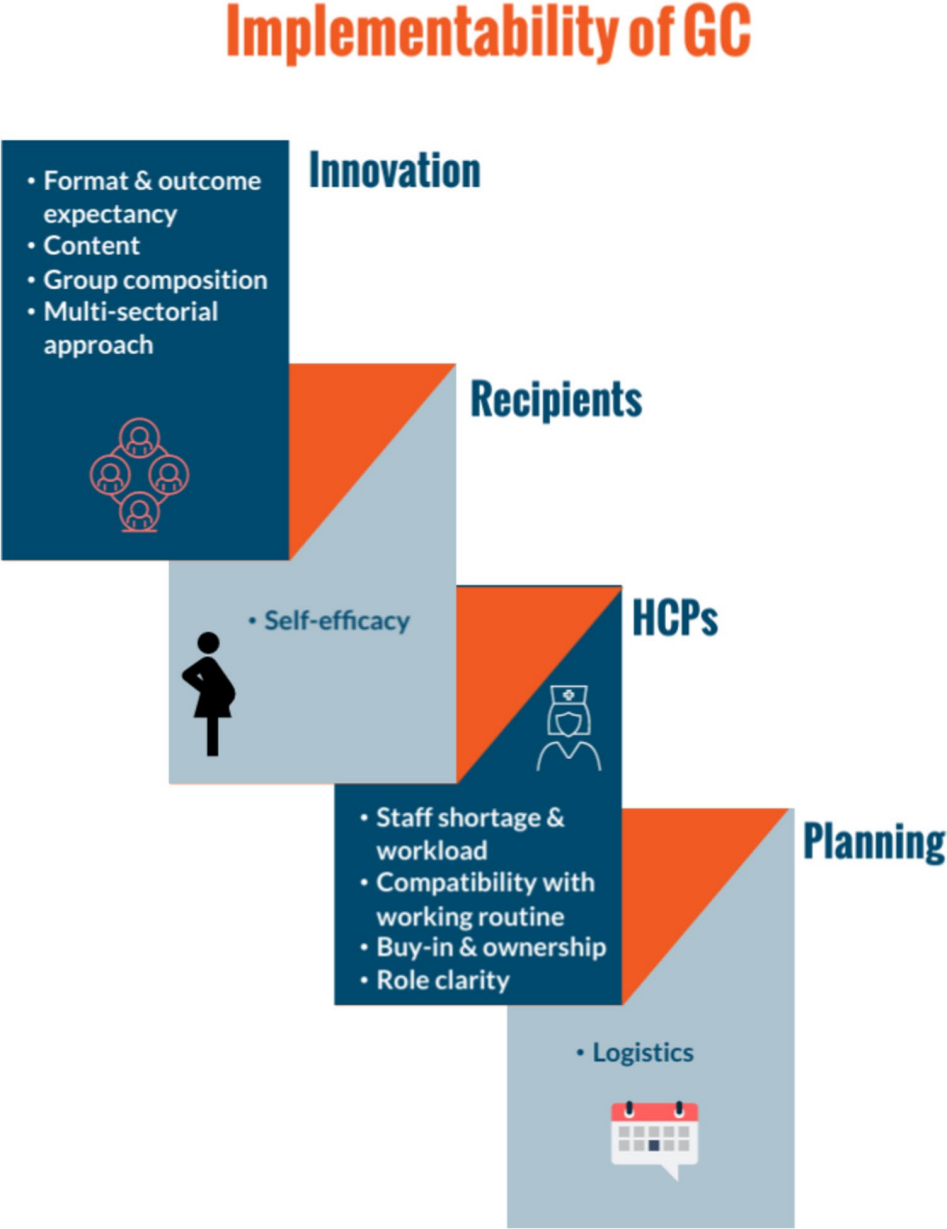
Implementability of GC

The sub-themes below were found to be related to the implementability of GC and as outlined in Fig. 1 they can be grouped into sub-themes that evolve around the innovation, the recipients, HCPs and around the planning of GC.

#### Implementability: innovation determinants

##### GC format and outcome expectancy

The overall perception of GC was positive across respondent categories. Educational and social aspects of the model were most frequently mentioned as potential implementation benefits. Policy makers, RGD managers and HCPs expected that implementing GC would improve parents’ health behaviours and enable the recognition of alarm signals for medical complications. Several HCPs expected improved pregnancy outcomes and a lower perinatal mortality as a result of the implementation of GC. One HCP adopted a more critical stance stating that GC will not solve the bigger socio-economic problems which are at the root of ill-health. The most salient advantage of GC named was increased information on pregnancy and parenting for recipients. Some recipients viewed GC as a recreational activity that can potentially offer relaxation, that can be *“gezellig”* (cosy, social), and that can enhance the relationship between both parents. One woman expressed concerns towards implementability: she stated that receiving bad news (e.g., miscarriage or disease) may be experienced more severely when in a group.

##### Content

To improve implementability of GC, parents suggested to discuss topics such as the developmental stages of their babies, nutrition of mother and child (including breastfeeding), family planning, unintended pregnancies, sex post-partum, mental health and self-care and practical issues. However, one woman anticipated that discussing traditional remedies with HCPs could be challenging due to conflicting views. In contrast, a RGD manager who used to work as HCP considered it crucial to discuss safe use of herbal medicine with particular attention for potentially harmful practices, such as hot steam baths that hamper wound healing, or eating *pimba (*white clay). On-site HCPs considered it particularly important to discuss breastfeeding and nutrition of mother and child. The majority of topics suggested by on-site HCPs would prepare parents for the postnatal period: arranging support for the first weeks postpartum, postnatal pain management, postnatal depression and caring for a new-born (e.g., naval care, constipation). External HCPs and policy makers emphasized the need to teach parents how to recognise alarm signals, so that calamities can be prevented. Additionally, HCPs suggested to pay more attention to hygiene, parenting skills, psychological and social needs of parents. Moreover, unintended pregnancies – frequently at a young age – appear to be very common. Hence, elaborate discussion of family planning during GC is warranted.

*“There really are women who say I didn’t know I had to come; they come with terribly swollen legs and a headache and they are already in, almost in a pre-eclamptic seizure. But they just don’t sense they should come or ring the alarm. So they need to get all that kind of information, they need to be made aware.”* Interview with HCP.

##### Group composition

Most recipients and HCPs thought that it is beneficial for fathers to join GC. However, a few women acknowledged that it would be easier to talk openly in the absence of men, enhancing implementability of GC. Several HCPs shared that concern. With regard to group composition in terms of age, SES and culture, community members and interviewed professionals were ambivalent. While they advocated for diversity, they also suggested that it would be challenging to implement groups where parents from the city and from the interior mix, and that it was important to keep ‘some sort of homogeneity’.

##### Multi-sectorial approach

Policy makers and HCPs emphasized that the diversity of determinants for neonatal and maternal health warrants a multi-sectorial approach. Numerous stakeholders that should be involved in the implementation of GC were named (e.g., Ministry of Health, Ministry of Social Affairs, *Bureau voor Openbare Gezondheidszorg* (BOG; public health office), Pan American Health Organization (PAHO), pediatricians, gynecologists, GPs, organization of midwives, social workers, psychologists), yet *how* these stakeholders should be involved remained unclear.

#### Implementability: recipients determinants

##### Self-efficacy

Recipients believed that they could learn how to measure their own blood pressure, or their baby's weight if well instructed, except for one woman who expressed low self-efficacy. She preferred the implementation of a GC model where HCPs are charge of all health assessments as she does not trust her own capabilities.

#### Implementability: HCPs determinants

##### Staff shortage and workload

At least one HCP at every implementation site and several HCPs from hospital settings mentioned that high workload and/or shortage of staff may affect the implementation of GC. A nurse from one of the implementation settings explained that sufficient staff needs to be available so that continuity is ensured also when one of the facilitators is sick, or on vacation.

##### Compatibility with working routine

On-site HCPs reflected on the potential impact the implementation of GC will have on their working routine. While one midwife expected a manageable increase of workload, other HCPs thought that the GC model is a more efficient way of working. They hypothesized that time can be saved when a group of parents receive information at once and that women might reach out to peers first before asking advice from HCPs.

Most on-site HCPs were eager to start the implementation of GC and were confident that it will be a success. However, one on-site GP doubted if GC will succeed in this environment due to the barriers to participation mentioned below. Concerns regarding sustainability were also raised by on-site staff; they do not wish to invest efforts in a temporary project but rather they aim to implement a different model of care that is sustained long-term.

##### Buy-in and ownership

On-site HCPs explained that buy-in from on-site RGD staff, including midwives, nurses and doctors was pivotal to implement GC but that the RGD management needs to be onboard, too. In fact, an implementation committee that includes RGD managers was already set up at the time of data collection. One midwife suggested that members from the RGD management should follow the GC training as this would increase understanding and support. While guidance from facilitators of the previous *SamenZwanger* pilot was welcome, a need for ownership was also voiced. A midwife explained: *“I also want to make it my own.”*

##### Role clarity

On-site HCPs were of the opinion that midwives are best suited to facilitate antenatal GC. However, nurses also demonstrated willingness to co-facilitate GC, whereas GPs expected less direct involvement and the assumption of an advisory role. One of the interviewed GPs admitted that he was shy and rather uncomfortable with the idea of facilitating group sessions. Health care coordinators, RGD management and external HCPs voiced a need to clarify roles and tasks within the GC_1000 project to strengthen implementability.

#### Implementability: planning determinants

##### Planning and logistics

For recipients it was crucial that dates are communicated in advance and that timing of GC sessions does not collide with work/school schedules. Community members added that many recipients attend church service on Sundays, rendering this an unsuitable time for GC sessions. A GP suggested to hold GC sessions during the weekend so that midwives would not neglect other duties. However, a midwife from the same setting reflected that good working conditions (such as flexibility regarding working hours, availability of material and space) are crucial for the implementation of GC and that conflict regarding working hours hindered the implementation of GC in a previous pilot. While some HCPs advised to organize GC during the midwives’ regular working time to minimize costs, another GP was of the opinion that no extra compensation for midwives was needed if GC was organised outside their regular work schedule because midwives would acquire new transferable skills through participation in this project. The timing of GC sessions is further complicated as a sufficiently spacious room in one setting is only available in the afternoons, when the clinic is more quiet. Furthermore, a policy maker suggested that the number of women that receive ANC at each RGD setting would be too small to form groups and hence several RGD clinics would have to liaise for recruitment.

#### Sustainability

##### Economic situation

Two policy makers from the Ministry of Health and the Staatsziekenfonds (SZF; State Health Insurance), Suriname’s largest health insurance, stated that in the currently strained economic situation, resources are sparce and hardly sufficient for curative care. All policy makers acknowledged that investments in preventative care are currently minimized and funding of GC through the SZF was ruled out. The respondent from the Ministry of Health explained that a sound budgeting plan is needed and effects on mortality and cost-effectiveness need to be demonstrated based on local data if the government was to support the implementation of GC. External HCPs doubted health insurances’ willingness to reimburse GC, and they also emphasised the need for a budgeting plan and scientific evidence.

*“I want to be convinced about the benefits. Because the moment I will have to spend money on it I want to see the benefits clearly otherwise I can spend the money in another way to have more benefits from the health sector. That is an honest answer.”* Interview with a policy maker.

On-site HCPs regarded lack of funding as the main implementation barrier and many HCPs reflected on the GC fee pregnant women/couples from two groups had to pay in the previous *SamenZwanger* project which led to restriction of women/couples who could afford it.

### Reach

**Fig. 2:**
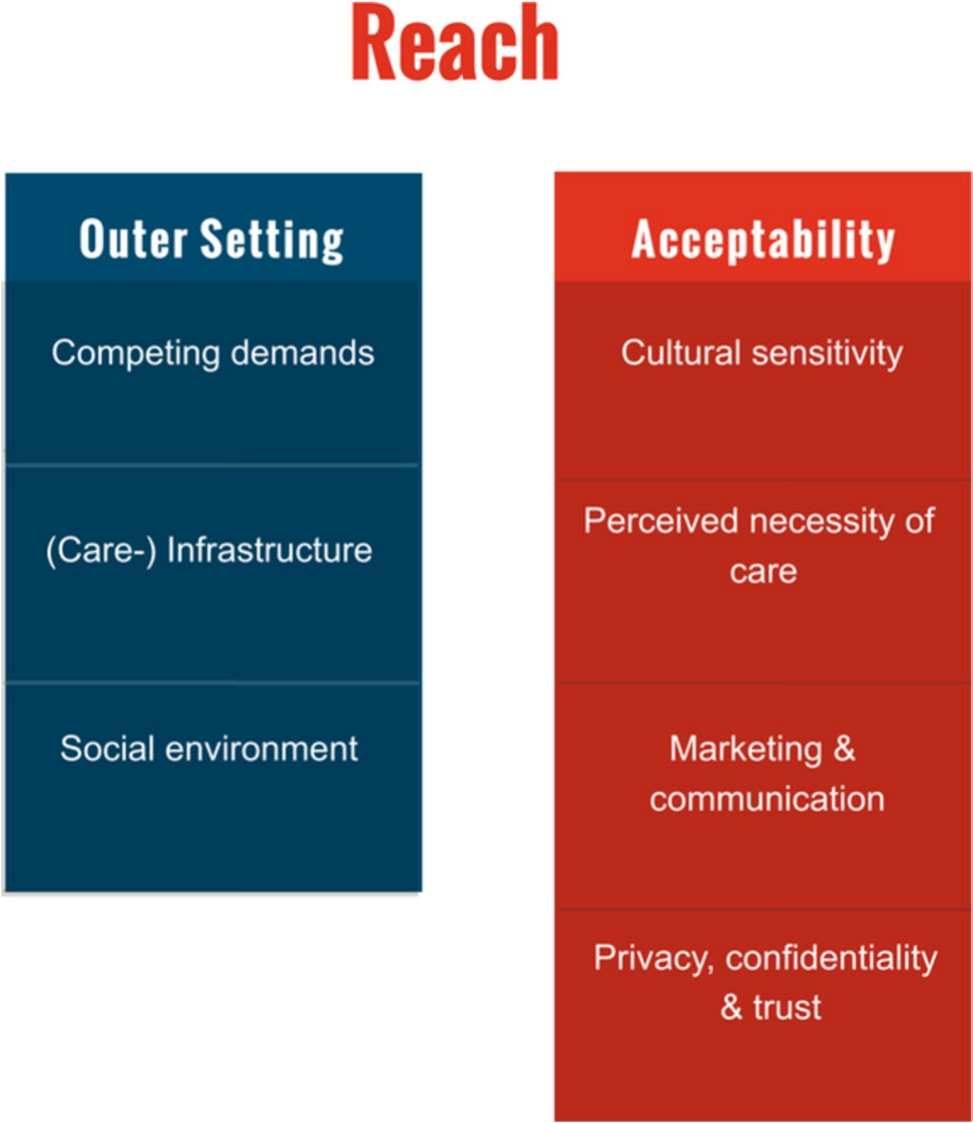
Reach

Below sub-themes are related to reaching and participation of the target population. Whereas some sub-themes describe barriers linked to the outer setting which hamper access to health care services in general, others evolve around willingness to participate in GC (see Fig. 2).

#### Reach: outer setting

##### Competing demands

Several parents, HCPs, and on-site HCPs reported long waiting periods after application for new health insurance cards. Without health insurance card, recipients need to pay for health care services, which many cannot afford. Multiple service-users denounced high costs for health care services and medication. Moreover, parents with economic stress are unlikely to prioritize GC when the time allocated to it could be used to generate income instead.

*“(…) someone who is struggling or stressed because she needs income will find it difficult to make time to come and do this [GC]. She prefers to think what she can do to get bread or food.”* Interview with community member.

##### (Care) infrastructure

Recipients, on-site HCPs and community members explained that poor infrastructure and long distances to health care facilities can be obstacles to seeking health care. Policy makers and HCPs pointed out that disparate access to health care is problematic. Women who live in the interior of Suriname frequently do not receive ANC at all, or they receive ANC from health assistants from the MMPHCS who are less trained in maternity care. Some women refuse referral to specialized care due to long distances. Lack of childcare is another barrier to attending ANC and PNC that was mentioned across respondent groups.

##### Social environment

Many respondents across categories speculated that partners and other family members may not permit women to attend GC. Recipients reasoned that disapproval from the social environment can stem from jealousy, or unwillingness to disclose private information. Moreover, reluctance towards novelty in general, including towards novel health interventions was mentioned in many interviews. The idea that a strained parental relationship can hamper GC attendance surfaced across respondent categories. Also, extramarital relations, multi-partnering and diversity in partner relations and.

family forms are not uncommon in Suriname and fathers may not want to be seen at GC with one of multiple partners, explained HCPs. Furthermore, groups where two women are pregnant of the same man can potentially lead to tensions.

#### Reach: acceptability

##### Cultural sensitivity

In Suriname pregnancies are often kept a secret (out of fear that others can negatively influence the health of mother and child). Interestingly, policy makers and HCPs thought that women may not want to join GC because of unwillingness to disclose their pregnancy, whereas women, their partners and community members did not mention it as a potential barrier. However, community members and recipients thought that some women may not be eager to participate in GC due to shame, embarrassment, or shyness. These attributes were frequently associated with origins from the interior of Suriname and low SES. Moreover, a policy maker thought that the cultural proscription for women to not leave their home for up to six weeks postpartum can interfere with GC attendance after birth.

Some women who live in remote areas of Suriname refuse to travel to Paramaribo to seek appropriate health care as they believe that they will lose their strong bond with nature in the city and get sick. As the various Surinamese regions are marked by ethnic and cultural diversity, GC facilitators should be familiar with the dominant local culture(s) and involve community members already in the planning phase, suggested a policy advisor.

*“Then you have to be careful, especially here in Suriname certain people are very sensitive when it comes to cultural matters, they feel easily stepped on the toes.”* Interview with HCP.

##### Perceived necessity of care

Recipients and community members explained that preventative care is frequently not considered necessary. Some multiparas think that they can rely on their previous experience and hence they regard ANC as futile. HCPs explained that mothers, aunts and female neighbours support women during pregnancy and give them advice. However, they also mentioned that their advice can be wrong and that the use of herbal medicine can prevent women from seeking professional health care. A recipient added that some parents are afraid of being criticised by HCPs.

*“At the outpatient clinic, the woman received instructions, but when she went back to her community she received other instructions, often wrong instructions. (…) If our pregnant women have a headache, it's not that they watched too much television or that they yelled at their children too much. There may be something seriously wrong.”* Interview with HCP.

Interview data across respondent categories indicates that sparse and late ANC attendance is common due to aforementioned practical barriers. Other reasons include not being aware of the pregnancy and cultural believes and practices.

*“Sometimes traditional practices make that women actually ask for help too late. For example in the Maroon community there is great resistance to caesarean sections, so if they hear that the child will have to be delivered with a caesarean section, they will first go looking for alternative solutions within their own community.”* Policy maker.

##### Marketing and communication

Recipients understood that GC is targeted at both parents and they speculated that single mothers and women with unintended pregnancies (especially teenage mothers) may feel excluded, or uncomfortable to attend. Recipients also explained that parents of lower SES may think that GC is aimed at parents of high SES. Moreover, interviewees from all respondent groups proposed to raise awareness of GC with a campaign using traditional and social media as well as religious leaders.

##### Privacy, confidentiality and trust

Respondents from all categories indicated that diminished privacy in GC will likely be a concern for recipients. Several HCPs rationalised that Suriname is a ‘small community’ and that therefore fear of judgement is high. While most recipients declared openness to sharing their experiences, they were also reluctant to discuss more private topics, such as marital conflict, miscarriages, mental health and sex during pregnancy. HCPs added that abnormal child development and domestic violence will likely be difficult to discuss in a group. Several respondents suggested that a safe environment facilitating open group discussions could be created by both facilitators and group members practicing self-disclosure and humour. HCPs also suggested to discuss all topics in a generalized manner and not at a personal level in order to ensure privacy and confidentiality.

*“I have to say that in Suriname we are quite suspicious of information that others want to hear from us and that we need to share.”* Interview with HCP.

In order to protect service-users’ privacy, it was advised to clarify during the intake what kind of information will be shared during GC sessions. One policy maker highlighted the need for facilitators to ask for permission prior to sharing any personal, or medical information with the group. On-site HCPs concluded that for privacy reasons it is important to host GC in a closed room and to continue offering one-on-one appointments next to GC sessions.

## Discussion

**Fig. 3:**
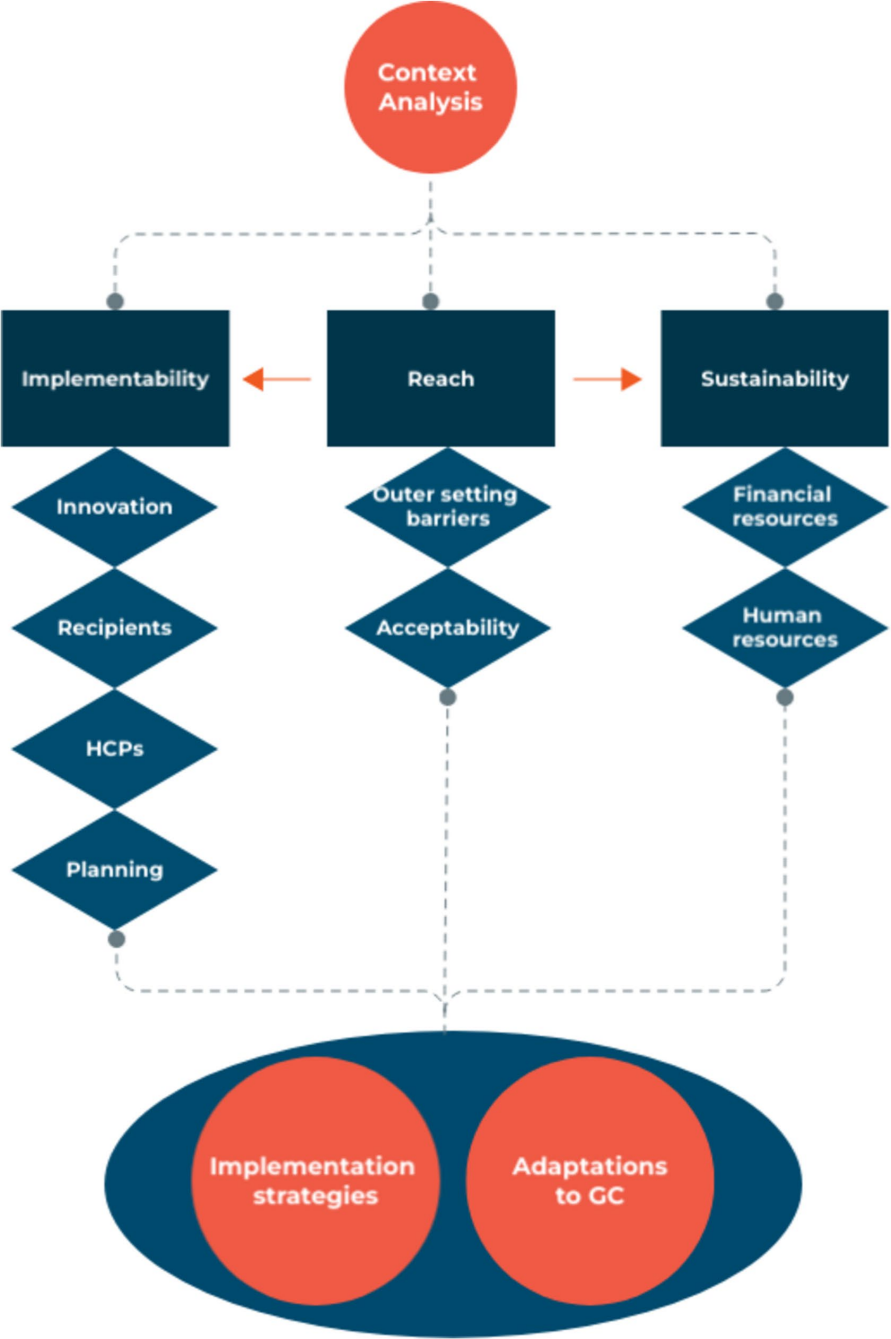
From context analysis to adaptations and implementation strategies

Findings of this context analysis describe factors that warrant adaptations to the GC model and the development of tailored implementation strategies prior to implementing GC in Suriname. Factors related to HCPs, to the innovation (GC), to recipients and to the organization impact the implementability of GC, while sustainability is hampered by sparse financial and human resources. Reach affects both implementability and sustainability; consistent participation allows for group cohesion and a sufficiently large number of recipients is needed to render GC cost-effective. Therefore, reach is the heart piece of sustained implementation of GC (see Fig. 3). Yet, outer setting and attitudinal barriers will likely affect reach.

The overall perception of GC was remarkably positive and tension for change (i.e., extent to which interviewees perceived the current model of maternity care as inadequate) was high, especially with regard to postpartum care. Nonetheless, strategies to reach the target population have to be developed and logistical and financial obstacles have to be overcome for successful implementation of GC in Suriname’s primary health care sector.

To implement GC sustainably, administrative buy-in at all levels is needed. As found in previous research [33–37] true buy-in—where financial and human resources are allocated to GC—demands ‘hard’ data on pregnancy outcomes and cost-effectiveness [33]. The generation of corresponding data is especially important in Suriname’s currently strained economic situation.

Sparse resources go hand in hand with logistical challenges. Adequate space and sufficient staff are needed to implement and sustain GC [33, 37, 38]. Interviewed HCPs described staff shortage all over the Surinamese health care sector and therefore the suggestion to organise GC outside of midwives’ working hours was made. However, a previous study reported that GC was discontinued when added on top of HCPs’ regular working schedule as signs of burnout became evident [33]. Overall, on-site HCPs in this study were optimistic about the implementation of GC. Yet, some concerns regarding efficiency, sustainability, and compatibility with the working routine were voiced. Moreover, a need to clarify the roles of all professionals and organizations involved in the project crystallized. When newly introduced, GC can disrupt the workflow which can cause tensions between colleagues [34, 37]. Therefore, aforementioned concerns of HCPs need to be addressed carefully.

Next to financial and logistical hurdles, recruitment challenges are frequently described in the literature [33, 37, 39, 40]. Our findings show that outer setting barriers and lacking acceptability of GC can hamper reach and participation of the target population. While poor (care) infrastructure and competing demands (e.g., lack of childcare/transport/health insurance, scheduling) are common practical barriers to GC attendance [33, 37, 40–42] and to accessing health care in general, attitudinal resistance is specific to GC. Concerns regarding trust and privacy in GC were not only reiterated across respondent groups in this study but they were also identified in multiple prior studies [37–40, 43–45]. The tightly-knit communities in the sub-urbs of districts Paramaribo and Wanica appear to act as catalysators for such apprehensions. Settings with similar social structures should be alert as they may encounter similarly accelerated concerns around privacy. One study identified five prerequisites to building trust in GC (vulnerability, communication, reciprocity, chemistry and atmosphere) and emphasized that the development of trust needs time [46]. Hence, when recruiting pregnant women/parents for GC – before parents have met other group members—it may not suffice to openly discuss privacy and confidentiality-related issues but the use of a confidentiality agreement may help overcome concerns at a time when trust had not time to develop, yet [46].

Furthermore, marketing of and communication around GC needs to be explicitly inclusive. Interviewed parents from our study explained that some recipients may be reluctant to join GC as it appears to target couples of high SES. Similarly, a previous study showed that the perception of GC targeting a specific sub-population can yield recruitment issues [37]. Moreover, reach can be jeopardized by discouragement from the social environment [39, 47] and by reluctance towards GC due to its novelty [33, 36, 39]. However, except for misoneism, reasons for discouragement from the social environment remain vague. To the best of our knowledge, strained parental relationships and multi-partnering specifically have not been identified as potential obstructions to GC attendance, yet. However, they might cause recruitment challenges in other settings with complex family constellations. Further reasons for unwillingness to participate in GC include cultural norms, such as non-disclosure of pregnancy and the view that preventative care is unnecessary. Non-disclosure of pregnancy has not been linked to GC recruitment issues, yet, although it is found in many cultures.

Cultural tailoring of the GC model and content is an essential step to increase reach [48]. In Suriname, the use of herbal medicine and traditional remedies is common [49]. For example, vaginal steam baths are a widespread ritual, especially postnatally. Yet, excessive use of steam baths is linked to ‘dry sex’ and quicker spread of sexually transmitted diseases [50]. As some traditional practices, such as vaginal steam baths, can be detrimental to health, they should be discussed in GC sessions in a manner that is respectful of the cultural heritage. On that account, it is crucial to consider the socio-political context when implementing GC in previously colonized countries; [48]especially in a multi-nation project such as GC_1000.

### Limitations

Response bias may have painted an overly positive picture of the perception of GC. We acknowledge this limitation and the fact that the prohibition of women to make critical remarks is so deeply rooted in the culture that it is hardly possible to overcome this limitation during a RQI. Due to a lack of actual experience with GC, it was challenging for interviewees to name advantages and disadvantages of the GC model, to suggest specific adaptations, or to foresee the community’s response to GC. However, as recognized in the CFIR addendum, [11] the investigation of anticipated rather than actual implementation outcomes lies in the nature of pre-implementation context analyses. We acknowledge that anticipated barriers and facilitators may differ from actual barriers and facilitators, and therefore evaluation efforts will continue throughout the implementation process. Moreover, interviews were conducted during the peak of the Covid-19 pandemic, which lead to a number of limitations: *(1)* several interviews were conducted online as traveling was not possible, *(2)* reliance on local researchers with less experience in qualitative research methods was inevitable (e.g., use of closed-ended questions), *(3)* during the interviews much time was allocated to discussing the impact of covid on GC. To overcome covid-imposed challenges, we discussed preliminary findings during elaborate online debriefings, visited implementation sites at a later stage and closely involved the leading Surinamese researchers for member checking of findings. Covid-related themes are not included in our findings as they are less relevant at the time of publication.

## Conclusion

Multi-layered contextual factors impact not only implementability and sustainability of GC, but also reach. Therefore – and in agreement with other implementation outcome taxonomies— [51, 52] we advise future researchers and implementors of GC to investigate not only determinants for implementability and sustainability, but also those factors that may hamper, or facilitate up-take. Practical, attitudinal and cultural barriers to GC participation need to be examined. As previously claimed, flexibility is a prerequisite when implementing GC in LMICs [53] but a comprehensive strategic plan that clearly outlines benefits and costs, roles of different professionals, location and scheduling as well as implementation strategies to enhance reach is equally important. Themes identified in this study will inspire the development of adaptations and implementation strategies at a later stage of the GC_1000 project.

### Supplementary Information

**Additional file 1.** 

## Data Availability

The data that support the findings of this study are not openly available due to reasons of privacy/ethics and are available from the corresponding author upon reasonable request. Data are located in controlled access data storage at Leiden University Medical Center and at the Foundation for Perinatal Interventions and Research in Suriname.

## Abbreviations

ANC: Antenatal Care
BOG: Bureau voor Openbare Gezondheidszorg
CB: Consultatie bureau/baby-well clinic
CFIR: Consolidated Framework for Implementation Research
GC: Group care
GC_1000: Group Care during the first 1000 days
GP: General Practitioner
HCPs: Health care professionals
MaMS: Committee for Maternal Mortality Suriname
MMPHCS: Medical Mission Primary Health Care Suriname
MMR: Maternal Mortality Ratios
NGO: Non-governmental organization
PAHO: Pan American Health Organization
PNC: Postnatal care
RGD: Regionale Gezondheidsdienst/Regional Health Service
SES: Socio-economic status
SD: Standard deviation
SZF: Staatsziekenfonds
WHO: World Health Organization
