# Supplementary material for: Anticipated benefits and challenges of implementing group care in Suriname’s maternity and child care sector: a contextual analysis
Source: BMC Pregnancy Childbirth. 2023 Aug 18;23:592. doi: 10.1186/s12884-023-05904-y (PMC10436662; doi:10.1186/s12884-023-05904-y)
Supplement: Supplementary file 1 — Additional file 1. [file 12884_2023_5904_MOESM1_ESM.docx]

Appendix 1

Interview guide for HCP

**Topic guide for HCP – Antenatal and Postnatal GC**

Opening Statement:

First of all, thank you for taking the time to talk to me today. Before we begin, I would like to quickly explain what you can expect and why we scheduled this interview.

My PhD research is embedded in a larger project with the aim to implement and sustain Group Care for pregnant women in 7 different countries. It is possible that different adaptations to the model are needed in these different settings. In order to find out what changes are needed, we are particularly interested in health care providers’ views, experiences and what is important to them. So, there are no wrong answers because we would like to listen to your opinion. This interview will take about one hour and it will be audio recorded. Before we start, do you have any questions?

| **Topic** | **Questions** |
| --- | --- |
| Start Questions | What is your professional background?  What is your role within the organization? |
| Current model of care & perception thereof | Can you describe the ANC trajectory for a pregnant person?   - Who provides antenatal care? Whom does she meet? - How many appointments? - How much time do you spend per consultation?   What aspects of ANC do you like/dislike? |
| Needs of women and babies | Can you describe your patient population? Who will be recruited for the groups?   - SES, Ethnicity, Age   Can you describe typical challenges of those pregnant women?   - Medical issues (HIV, family planning…) - Practical (transport, financing…) - Psycho-social (support…) - Culture-specific (language, etc.) |
| Perception of GC | What do you know already about GC?  VIDEO HERE if needed  What do you like/dislike about GC?   - Advantages/disadvantages for: you, the organization, women? - Compared to the current antenatal care? - Interactive learning methods, such as throwing a ball, introducing the person sitting next to you, self-assessment |
| Personal involvement and motivation to implement GC | What is your role within the implementation of GC?  How did you get involved in the implementation of GC?   - Did you decide it yourself? Why? Did someone ask you? - What motivates you?   How was the decision made to implement Group Care in your setting?   - Who participated in the decision-making process? - What were the reasons for this decision? - How was the decision communicated to you?   How will GC affect you?   - working situation/Professional development/career? Work/life balance? |
| Implementation process/planning | Can you describe the plan for implementing Group Care within your setting?   - How detailed is it? Who knows about it? - How will Group Care be integrated into the current organization of antenatal care in your setting? Will the intervention replace or compliment a current program or process? - How will women be recruited to participate in the Group Care? - How will it be possible in times of COVID? Is blended care an option? |
| Implementation barriers/facilitators - adaptations | In your view, how can successful implementation be ensured? What do you need?   - Changes to the GC model - Changes to the organization - Who needs to be onboard - Changes in health care system - In times of COVID   What might hinder the implementation of GC?   - Policies: local/national regulations or guidelines (e.g., reimbursement) - General health care system in your country - Socio-cultural factors - Competing projects - Staff |
| Self-efficacy | What will be challenging for you as a facilitator?   - Recruitment - Leading group discussion - Certain topics   What do you need to resolve those challenges? |
| Inner setting: organizational structure | Can you describe the working climate in this organization?   - Relationship with colleagues and leaders - Motivation of staff   Can you describe the values/priorities of your organization?   - Hierarchical, entrepreneurial, competitive, client-centered   How well does Group Care fit into this organization?   - Think of values, norms, management style   So far, how have the managers of your organization been involved in the implementation of GC?   - Who are these leaders? How do attitudes of different leaders vary? - What kind of support have they given you? Example? - What kind of support or actions do you expect from leaders in your organization to help make the implementation of Group Care successful? |
| Stakeholders | When you need to get something done or to solve a problem, who are your "go-to" people within the organization? Example?  Who do we need to get on board for the implementation of GC? Why?  Who do you ask if you have questions about GC?  Who outside of your organization will help with implementation of GC?   - What is their role? What do you expect? |
| Recommendations - closing | How can GC be made more attractive for you and other facilitators?  Do you have any suggestions for the implementation of GC?  Thank you for participation |

Appendix 2

**Topic guide for Women with NO CP-experience**

Opening Statement:

First of all, thank you for taking the time to talk to me today. Before we begin, I would like to quickly explain what you can expect and why we scheduled this interview.

My PhD research is embedded in a larger project with the aim to implement and sustain Group Care for pregnant women in 7 different countries. It is possible that different adaptations to the model are needed in these different settings. In order to find out what changes are needed, we are particularly interested in the women’s views, experiences and what is important to them. So, there are no wrong answers because we would like to listen to your opinion. This interview will take about one hour and it will be audio recorded. Before we start, do you have any questions?

| Topic | Questions |
| --- | --- |
| Start Questions | Age  Age of child/week of pregnancy  Family situation: Do you already have children? Partner?  Living Situation (Where? How? Who?)  Profession/daytime activity  Education |
| Personal Experiences with antenatal care | How did you find out that you were pregnant? What happened then?  Can you describe your experience with ANC?   - Where did you go first? - How did you feel during the consultations? - How was your relationship with the HCP? - What did you like? - What could be better? What did you dislike? What was missing? |
| Health info | What did you learn about the health aspects of pregnancy/delivery and child health?   - Examples, what was new to you?   How/where did you learn about it?   - Where do you get information about family planning? |
| Traditions/Rituals | What role do /did traditional midwives/healers play during and after your pregnancy?   - What about alternative medicine?   Can you describe events/rituals/traditions…  …at beginning of pregnancy?  …end of pregnancy?  …at birth?  …after birth?   - Who do you meet? What is expected? |
| Needs | Can you describe how you experienced the beginning of your pregnancy?  Can you describe how you experienced the end of your pregnancy?  Can you describe how you experienced the first weeks after giving birth?   - What was important to you? - What was challenging? - What did you worry about? - What questions did you have? - What were you missing? - Physically, mentally, socially   Can you describe any challenges that other women/families faced during/after pregnancy?   - How did you/they cope with these challenges?   In your opinion, what is especially relevant for pregnant women/young mothers and their babies in your community?  How are your expenses for ANC covered?   - Health insurance? Free? government? Family/partner? |
| Beliefs & attitudes | Do you think that it is beneficial for pregnant women to attend antenatal care? Why/why not? |
| Video | |
| General impression of GC | What do you think about this form of care?   - What do you like/dislike? - In your opinion what are the benefits of GC? - What are your concerns about this form of care? - How do you feel about GC in times of **COVID**? - And about online GC? |
| Perception of core elements | Can you describe how you would feel receiving individual health assessments in the group space?   - Fetal heartbeat, position of baby, blood sample   Can you describe how you would feel when taking your own health assessment and recording it? e.g., blood pressure  How you would feel when sharing your own experiences/questions in a group/When listening to others' experiences and questions? |
| Topics/format | What questions would you like to ask in the group discussion?   - What would you like to learn about?   Which topics would you feel uncomfortable talking about?   - What would make you feel more comfortable to share your experiences and questions?   Can you think of any pregnancy-related topics that are generally not talked about openly?  Would you prefer partners to attend or not attend the sessions? Why? |
| Obstacles | Can you think of any reasons that might prevent women from attending GC?   - Can you think of any reasons why women might not *want* to participate in GC? - Can you think of any solutions? |
| Social support | What would your friends/family/ partner say if you told them that you will attend GC? |
| Suggestions | Would you attend GC? Why/why not?   - What would make GC more attractive for you? When would you attend?   Would you like to add anything? |
| Closing | **Thank for participation and hand debriefing sheet** |

Appendix 3

**Topic guide for outer setting stakeholders**

- Estimated time: 30-40minutes

- introduction about the research, and more specific about the setting

| **Topic** | **Questions** |
| --- | --- |
| Start Questions  3A | -By who/which organization are you employed?  -What is your title within your organization?  -What are your main responsibilities? (before implementation of GC)  -What’s your professional background? |
| Vulnerable pregnant women | Can you describe the needs of (vulnerable) pregnant women in your region? |
|  | **Vignette - video** |
| Personal view and knowledge about GC by Health Care Manager | -What’s your first impression of Group Care after seeing this video? (4A)   - dvantages/disadvantages? Compared to current antenatal care? - what do you know about the evidence based of GC?   - Can you tell me what you know about experiences with group care? - Other organizations? - * Participants? |
| GC within organization: WHY | - How will group care affect your organization?  - How do you think GC can meet the needs of the pregnant women in your organization?  - Can you think of any regulations that might affect the implementation of GC?  * local/state/national?  * reimbursement? …  - What aspects of the general health care system in your country will influence the implementation of GC in that setting? (Facilitate or impede?)  *- Is Group Care seen as a priority in your organization? Can you give some examples that reflect this? (3D.c)*  *- How do you prioritize the implementation of GC in that setting? Can you also give some examples? (3C)* |
| GC within organization:  Decision process | - How is your organization involved in the implementation of Group Care? (if applicable)  * What is your role within this implementation project?  * How were you/your organization involved in the decision process?  - Why do you consider your organization important to be involved in the implementation and organization of GC?  - Can you tell me how you/your organization could support the implementation/organization? |
| GC within organization: HOW | - Can you describe how Group Care will be integrated into the daily work (in your organization)?  - What is your plan for getting the word out about Group Care?  * How will you inform people about the existence of group care?  - How will you recruit women for attending GC? (if applicable)  ** What materials/modes/venues do you plan to use? For example e-bulletin boards, emails, brochures?  * What process do you plan to use to communicate? For example, going to staff meetings, talking to people informally? * reaching the women / motivating to attend/ …* |
| Key stakeholders | - Who are other key influential individuals to get on board with this implementation of Group Care? (5B.a)  * To encourage individuals to use the intervention?   * To help with implementation? |
| Closing | -What would make GC more attractive for you as a partner in de implementation of GC?  - Do you have any other suggestions for the implementation of GC?  - Thank for participation |
|  |  |
